# The Feet in Winter-Time

**Published:** 1859-11

**Authors:** 


### THE FEET IN WINTER-TIME.

No person can be well long, whose feet are habitually cold; while securing for them dryness and warmth, is the certain means of removing a variety of annoying ailments.

The feet of some are kept more comfortable in winter if cotton is worn, while woolen suits others better. The wise course therefore is for each one to observe for himself, and act accordingly.

Scrupulous cleanliness is essential to the healthful warmth of the feet; hence all, especially those who walk a great deal out of doors during the day in cold weather, should make it a point to dip both feet in cold water on rising every morning, and let them remain halt ankle deep, for half a minute at a time, then rub and wipe dry, dress and move about briskly to warm them up. To such as cannot well adopt this course from any cause, the next best plan is to wash them in warm water every night just before going to bed, taking the precaution to dry them by the fire most thoroughly before retiring; this, besides keeping the feet clean, preserves a natural softness to the skin and has a tendency to prevent and cure corns, Many a troublesome throat affection, and many an annoying headache will be cured if the feet are kept always clean, warm, soft and dry.

The moment the feet are observed to be cold, the person should hold them to the fire, with the stockings off, until they feel comfortably warm. One of the several decided objections to a furnace heated house, is the want of a place to warm the feet, the registers being wholly unsuited for that purpose. Our wealthy citizens do themselves and their families a great wrong if they fail to have one room in the house, free for all, where a fire is kept burning from the first day of October until the first day of June, on a low grate, on a level with the hearth, after the pattern of Andrews & Dixon, of Chestnut street, Philadelphia; for the closer the fire is to the hearth in a grate, or to the floor in a stove, the more comfortable is it, and the less heat is wasted. This is one of the delights of the good old fashioned wood fires, the very thought of which carries so many of us away to the glad scenes of childhood and early homes. It ought to be known in New York, where hard or anthracite coal is burned, that with one of the grates named, filled with hard coal and a few pieces of Liverpool or cannell put on top, nearly all the advantages of a wood fire are secured, at least as far as cheerfulness, comfort and warmth are concerned.

Some feet are kept cold by their dampness from incessant perspiration, in such cases cork soles are injurious, because they soon become saturated, and maintain moisture for a long time.—Soak a cork in water for a day or two and see. A better plan is to cut a piece of broad-cloth the size of the foot, baste on it half an inch thickness of curled hair, wear it inside the stocking, the hair touching the sole; remove at night and place before the fire to dry until morning. The hair titilates the skin, thereby warming it some, and conducts the dampness to the cloth.

Scrupulous cleanliness of feet and stockings, with hair soles, are the best means known to us of keeping the feet warm when they are not cold from decided ill health. A tight shoe will keep the feet "as cold as ice," when a loose fitting one will allow them to be comfortably warm. A loose woolen sock over a loose shoe will maintain more warmth than the thickest soled tight fitting boot. Never start on a journey in winter, nor any other time, with a new shoe.

